# Pharmacology, Medicinal Chemistry, and Therapeutic Potential of Imidazoline Receptor Ligands

**DOI:** 10.1002/cmdc.70350

**Published:** 2026-07-07

**Authors:** Maria‐Eleni Kouridaki, Mercè Pallàs, Carmen Escolano

**Affiliations:** ^1^ Laboratory of Medicinal Chemistry Department of Pharmacology Toxicology and Medicinal Chemistry Faculty of Pharmacy and Food Sciences University of Barcelona Barcelona Spain; ^2^ Pharmacology Section Department of Pharmacology Toxicology and Therapeutic Chemistry Faculty of Pharmacy and Food Sciences University of Barcelona Barcelona Spain; ^3^ Centro de Investigación en red Enfermedades neurodegenerativas (CIBERNED) Instituto de Salud Carlos III Madrid Spain; ^4^ Institute of Biomedicine (IBUB) University of Barcelona Barcelona Spain

**Keywords:** analgesia, imidazoline receptor, ligand, medicinal chemistry, neurodegeneration, neuroprotection

## Abstract

The imidazoline receptor (IR) system, comprising the I_1_R, I_2_R, and I_3_R subtypes, consists of binding sites involved in cardiovascular, metabolic, and neurological disorders. This review updates the 2004 compilation by Dardonville and Rozas on IR ligands, emphasizing promising ligands, subtype selectivity, and pharmacological profiling. Representative ligands for each subtype are analyzed to highlight key pharmacological aspects, including affinity, selectivity, and functional activity, integrating findings from preclinical and clinical studies. Critical molecular targets such as Nischarin/IRAS for I_1_R and MAO‐B–associated sites for I_2_R are discussed in the context of ligand design and CNS penetration. I_1_R‐selective ligands, exemplified by rilmenidine, show improved selectivity over α_2_‐adrenoceptors and exhibit antihypertensive, metabolic, and neuroprotective effects. I_2_R ligands display neuroprotective, anti‐inflammatory, and analgesic activities, with CR4056 progressing to Phase II trials. PET imaging with [^11^C]BU99008 has validated I_2_R upregulation as a biomarker for neurodegeneration. Overall, the IR system presents therapeutic opportunities: I_1_R for cardiovascular and metabolic disorders, I_2_R for pain and neurodegeneration, and I_3_R for diabetes. Continued ligand optimization and receptor characterization are essential for clinical translation.

## Graphical abstract

Building on the excellent review article by Dardonville and Rozas (2004), this paper summarizes the most representative ligands of imidazoline receptors (IR; I1, I2, and I3) and updates their pharmacological profiles. Although not exhaustive, it provides a solid starting point for understanding the significance of I2 IRs as promising therapeutic targets.

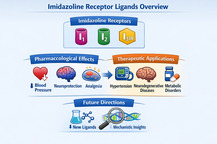

## 1 Introduction

This review provides a comprehensive and up‐to‐date structural and pharmacological evaluation of imidazoline receptor (IR) ligands, following the rigorous analytical framework by Dardonville and Rozas [1]. That seminal work classified ligands acting at IR according to their structural scaffolds, binding affinities, and pharmacological relevance. Over the past two decades, progress has been made in identifying IR‐associated molecular targets, developing selective ligands, refining structure–activity relationship (SAR) principles, and validating therapeutic applications, particularly in central nervous system (CNS) and metabolic disorders. Numerous diseases still lack effective therapeutic options, or available treatments provide only limited efficacy. Consequently, considerable efforts by the scientific community are required to identify new therapeutic targets whose modulation by selective, high‐affinity ligands elicits beneficial physiological responses. In many cases, disease pathophysiology involves complex networks of molecular targets, and one of the major challenges in drug discovery is to modulate these networks in a balanced manner. In this context, extensive research efforts over the years have linked IR to a wide range of pathophysiological conditions, highlighting them as promising therapeutic targets. In 2020, the leading experts in IR, P. Bousquet, A. Hudson, J. A. García‐Sevilla, and J.‐X. Li, revisited the imidazoline system from a past, present, and future perspective, compellingly highlighting its outstanding therapeutic promise. Their analysis underscored how modulation of this system with highly affine and selective ligands may open new avenues for the treatment of a broad spectrum of clinically relevant diseases [2]. Adopting the structural logic and comparative rigor of Dardonville and Rozas (2004), this work systematically maps the affinity, selectivity, pharmacological profiles, and therapeutic potential of key imidazoline ligands, organized by receptor subtype. Notably, IRs are divided into three subtypes (I_1_R, I_2_R, and I_3_R) according to their affinity for radioligands and constitute molecular entities with little or no structural information available, which significantly hampers their characterization. In this article, ligands are classified according to the receptor subtype they interact with, and selected representative compounds are discussed, without the aim of providing an exhaustive survey of all reported ligands, but only the most representatives.

## 2 Pharmacology and Therapeutic Scope of I_1_R Ligands

The I_1_ imidazoline receptor (I_1_R), labeled by [^3^H]clonidine and [^3^H]idazoxan, has attracted considerable interest as a potential therapeutic target in cardiovascular, metabolic, and neuropsychiatric disorders, and several selective ligands have been reported [3]. Despite the lack of definitive molecular identification, I_1_Rs have been closely associated with Nischarin/Imidazoline Receptor Antisera‐Selected protein (IRAS) and, alternatively, with the sphingosine‐1‐phosphate receptor 3 (S1P_3_), a G‐protein‐coupled receptor. Nischarin has been shown to mediate I_1_R‐like activation of the mitogen‐activated protein kinase (MAPK) pathway. In PC12 cells, rilmenidine (10 μM, 15 min), a centrally acting I_1_R‐selective antihypertensive agent, significantly increased phospho‐MAPKp42/44, an effect attenuated by antisense oligonucleotides targeting Nischarin. Immunoblotting revealed a 210 kDa band in native PC12 and a 190 kDa band in NIH3T3 cells transfected with IRAS cDNA with immunofluorescence, confirming partial membrane localization. In vivo, Nischarin expression was localized to the ventrolateral medulla of neonatal rats. These regions are essential for autonomic control and respiratory drive, aligning with prior observations of preserved inspiratory flow under dexmedetomidine, a compound with I_1_R activity. An alternative hypothesis suggests that I_1_R may be structurally related to or form heteromers with S1P_3_ receptors. In PC12 cells, both [^3^H]clonidine, an α_2_‐AR/I_1_R agonist, and [^3^H]lysophosphatidic acid, displayed specific binding displaced by S1P, whereas moxonidine showed weaker displacement. In transfected HEK293 cells, S1P_3_‐mediated Ca^2+^ influx was evoked by S1P, clonidine, and notably moxonidine, with no response in native HEK293, suggesting receptor‐specific activation [4, 5, 6, 7].

**FIGURE 1:**
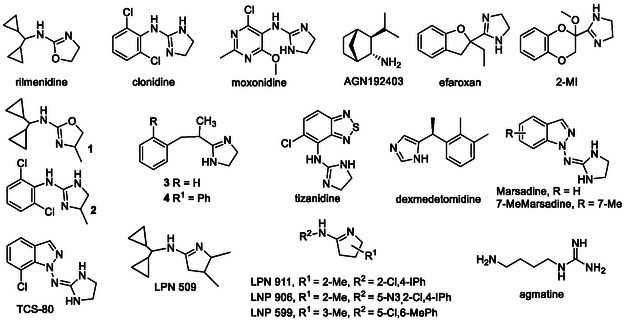
Representative I_1_R ligands.

Selected I_1_R ligands are listed and the structure depicted in Figure 1.

### 2.1 Moxonidine

Moxonidine displayed high affinity for human I_1_R (pK_i_ = 8.38) and, in bovine tissues, yielded pK_i_ values of 8.61 (I_1_R), 7.11 (α_2_‐AR), and 5.01 (I_2_R), comparable to benchmark selective ligands such as AGN192403 (pK_i_ = 7.46 at I_1_R; 4.66 at α_2_‐AR) [8]. Selective activation of I_1_R in the rostral ventrolateral medulla underlies the sympathoinhibitory and antihypertensive effects of moxonidine, supported by studies where efaroxan, a I_1_R antagonist, reversed its hypotensive action more effectively than 2‐methoxyidazoxan, an α_2_‐AR antagonist [9, 10, 11, 12, 13]. Neurochemical lesioning of rostral ventolateral medulla noradrenergic terminals with 6‐hydroxydopamine, a neurotoxin that selectively depletes noradrenaline, reduced moxonidine's efficacy by up to 60%, highlighting its dependence on intact noradrenergic input. Peripheral sympathoinhibition was explored in pithed Wistar rats, where moxonidine dose‐dependently increased cardioaccelerator sympathetic outflow, increasing diastolic pressure from 33 ± 4 mmHg to 61 ± 9 mmHg at 10 µg/kg/min without affecting heart rate [14]. At 3 µg/kg/min, this effect was abolished by the α_2_‐AR antagonist rauwolscine, whereas the I_1_R antagonist (pK_i_ of 7.46) AGN192403 was ineffective when administered alone, indicating a predominant role of α_2_‐ARs at this dose. At 10 µg/kg/min, partial reversal by rauwolscine and full abolition with the combination of rauwolscine and AGN192403 revealed coactivation of α_2_‐ARs and I_1_Rs. The inability of AGN192403 to block the response is consistent with the higher I_1_R affinity of moxonidine and simultaneous α_2_‐AR activation. In the same model, moxonidine suppressed frequency‐dependent tachycardic responses, confirming a prejunctional mechanism, again requiring both rauwolscine and AGN192403 to abolish its effect, supporting a cooperative role of α_2_‐AR and I_1_R. High doses of moxonidine in spontaneously hypertensive rats revealed an α_2A_‐AR blockade. Even under I_1_R blockade with AGN192403 (3000 µg/kg), moxonidine at 10 µg/kg/min produced significant diastolic and systolic pressor responses (from 43 ± 8 mmHg to 73 ± 11 mmHg and 76 ± 4 mmHg to 104 ± 6 mmHg, respectively) without HR changes, indicating peripheral sympathoinhibition can persist independently of I_1_R signaling and highlighting the dose‐ and condition‐dependent receptor recruitment that characterizes moxonidine [15].

### 2.2 Rilmenidine

Rilmenidine favors I_1_R‐mediated pathways while minimizing α_2_‐AR related effects [16, 17, 18, 19]. Radioligand data indicate an I_1_R/α_2_‐AR affinity ratio of∼30, surpassing clonidine (3.8) and comparable to moxonidine (33), explaining its reduced sedation and dry mouth relative to α_2_‐AR agonists. Nonetheless, α_2A_‐AR engagement is still necessary for full cardiovascular responses, as I_1_R activation in the rostral ventolateral medulla modulates sympathetic tone upstream. In vivo, rilmenidine's central sympathoinhibitory profile has been defined through baroreflex and sympathetic nerve activity–arterial pressure (SNA‐AP) studies: high‐dose rilmenidine (250 µg/kg) narrowed the SNA response range (89.6% ± 2.9% to 50.4% ± 7.9%), lowered the SNA lower asymptote (13.5% ± 3.0% to 2.7% ± 1.5%), increased the SNA–AP intercept (57.1 ± 3.8 to 78.2 ± 2.7 mmHg), and reduced the slope of the SNA–AP relationship from 0.82 ± 0.08 to 0.51 ± 0.07 mmHg/%. Weak peripheral vasoconstriction (20 mmHg AP increase without SNA change) did not impair its central hypotensive effect (–19.8 ± 2.2 vs. –26.4 ± 5.3 mmHg). Additionally, rilmenidine inhibits the renal Na^+^/H^+^ antiporter, promoting natriuresis and avoiding Na^+^/water retention, while subtly modulating baroreflex gain and input pressure in anesthetized rats, preserving postural cardiovascular reflexes during therapy [20]. Rilmenidine inhibits L‐type Ca^2+^ currents in cardiomyocytes via an I_1_R–phosphatidylcholine‐phospholipase C–diacylglycerol (DAG)–protein kinase C (PKC)–serine/threonine protein phosphatase pathway, independent of protein kinase A/nitric oxide signaling, with effects diminished in spontaneously hypertensive rats [21]. It further demonstrates proapoptotic and antiproliferative activity in K562 leukemia cells, synergizing with doxorubicin, and modestly attenuates morphine withdrawal at high doses in vivo despite lacking cAMP modulation in vitro. Activation of I_1_R has also been implicated in reducing basal ganglia–associated rigidity in motor dysfunction models [22]. Mechanistically, rilmenidine triggers rostral ventolateral medulla neuronal MAPK (p42/44) phosphorylation through a phosphatidylcholine‐specific phospholipase C pathway, distinct from α_2_‐AR signaling. This effect is abolished by I_1_R antagonists or extracellular signal‐regulated kinases 1 and 2 (ERK1/2) inhibition, supporting MAPK involvement downstream of I_1_R, with antisense downregulation of Nischarin confirming its role as a functional I_1_R linked effector. Methylation of rilmenidine, led to compound **1**, that displayed reduced I_1_R affinity (pK_i_ = 6.44 vs. 7.95) while still significantly lowering α_2_‐AR activity.

### 2.3 Clonidine

Clonidine shows moderate affinity for human platelet I_1_R (pK_i_ = 7.26 under norepinephrine masking), alongside higher affinity for α_2_‐AR (K_i_ = 2.0 ± 0.13 nM for α_2A_‐AR, 2.8 ± 0.48 nM for α_2B_‐AR, 2.7 ± 0.18 nM for α_2C_‐AR), resulting in an I_1_R/α_2_‐AR selectivity ratio of approximately 3.8. Its dual profile makes it a useful benchmark for comparing selective I_1_R ligands [23]. Methylation of the heterocyclic ring of clonidine led to compound **2** with increased I_1_R affinity (pK_i_ = 9.03) and markedly reduced α_2_‐AR binding (up to 224‐fold at α_2A_‐AR), making it the first imidazoline hypotensive agent without vasoconstrictive effects [24]. When an ethylene chain links the phenyl and the imidazoline ring and a methyl group led to compound **3** with enhanced I_1_R selectivity by interacting with a putative “methyl pocket” on the receptor [25]. Stereospecific changes produced dramatic shifts: for example, *R*‐**3** favored I_1_R engagement with an I_1_R/I_2_R ratio of 186 and a pronounced enantiomeric preference (eudismic ratio 5888 for the *S*‐**3**) [26]. Compound **4** with an *ortho*‐phenyl group shifted from I_1_R antagonism to agonism, likely because of new π–π interactions, and was endowed with antineuropathic and antiproliferative activity [27]. Functionally, clonidine reduces sympathetic nerve activity in a dose‐dependent fashion (2–5 μg/kg i.v.), with high doses (5 μg/kg) elevating the intercept of the peripheral arc (Δ = +21.5 mmHg) without slope modification, consistent with peripheral α_2_‐AR mediated vasoconstriction. Under these conditions, the operating‐point AP (81.3 mmHg) exceeded the extrapolated central‐only AP (70.7 mmHg; *P* < 0.05), reflecting partial offset of I_1_IR driven sympathoinhibition by peripheral α_2_‐AR engagement. In experimental hypertension, clonidine (30 μg/kg i.v.) attenuated chronic sinoaortic denervation‐induced hypertension comparably to moxonidine (100 μg/kg), whereas guanabenz, a selective α_2_‐AR agonist, was ineffective, indicating I_1_R involvement despite clonidine's mixed profile. In neurobehavioral models, clonidine (20 μg/kg) suppressed cue‐induced cocaine‐seeking reinstatement in rats, with lower doses inactive, suggesting dual I_1_R/α_2_‐AR mechanisms. I_1_R‐selective agents such as moxonidine and rilmenidine achieved similar antirelapse effects without the sedation classically associated with clonidine, which is linked to α_2_‐AR mediated suppression in the locus coeruleus [28].

### 2.4 Tizanidine

Tizanidine an α_2_‐AR/IR ligand, exhibits moderate affinity for human platelet I_1_R (pK_i_ = 7.55), with weaker affinity for α_2A_‐AR (55.7 ± 17.7 nM), α_2C_‐AR (120.0 ± 48.0 nM), and α_2B_‐AR (178.0 ± 84.0 nM). Functionally, tizanidine suppresses spinal mono‐ and polysynaptic reflexes in vivo (1–2 mg/kg s.c. in traction tests; 100 μg/kg i.v. for reflex models), an effect resistant to yohimbine (50–100 μg/kg i.v.), consistent with IR–driven pharmacodynamics [29]. Clinically, tizanidine is an approved, centrally acting antispasticity drug (marketed as Sirdalud or Zanaflex) used to reduce muscle spasm, cramping, and tightness in multiple sclerosis and spinal‐cord injury [30, 31]. Tizanidine's centrally mediated motor‐suppressive and antirigid effects have been validated in a Parkinsonian rigidity model, where i.p. doses of 1 and 3 mg/kg significantly reduced EMG‐measured hindlimb rigidity in reserpine‐treated mice. Of note, α_2_‐AR antagonists such as yohimbine, though capable of reducing rigidity, did not reverse the antirigid action of tizanidine, confirming a functionally IR‐predominant mechanism.

### 2.5 Dexmedetomidine

Dexmedetomidine displays high affinity for α_2_‐ARs (pK_i_ = 8.57) and lower affinity for I_1_Rs (pK_i_ = 4.84). Clinically, it is an approved α_2_‐AR agonist marketed as a sedative and analgesic for intensive care and procedural sedation (trade names Dexdor and Precedex) [32, 33]. Its imidazoline motif confers partial I_1_R agonism in relevant tissues, suggesting conformation‐dependent signaling mechanisms where tissue‐specific effectors, rather than high receptor affinity per se, determine its pharmacodynamic footprint. In guinea pig sinoatrial node cells, dexmedetomidine produced a concentration‐dependent negative chronotropic effect, reducing spontaneous firing by ˜13% at 10 nM and up to ˜33% at 1 µM, via suppression of the hyperpolarization‐activated current (If), an effect reversed by the mixed α_2_‐AR/I_1_R antagonist efaroxan but unaffected by yohimbine, confirming I_1_R involvement rather than α_2_‐AR pathways. In the CNS, dexmedetomidine increased p‐ERK1 and p‐ERK2 expression in hippocampal slices through I_1_R coupled signaling, while in the ventrolateral medulla of newborn rats, dexmedetomidine preserved inspiratory flow despite sedation, consistent with I_1_R recruitment and supported by the detection of Nischarin as a putative I_1_R‐protein [34].

### 2.6 Marsanidine

Marsanidine a potent and centrally acting antihypertensive compound possesses a pK_i_ of 7.85 for α_2_‐AR and a markedly lower affinity for I_1_R (pKi = 4.26). 7‐Memarsanidine is a mixed α_2_‐AR/I_1_R agonist with superior renal and cardiovascular effects that have been attributed to its dual‐receptor targeting, pointing that hybrid ligands may yield synergistic or potentiated effects, mitigating side effects linked to pure α_2_‐AR stimulation [35].

### 2.7 TCS‐80

TCS‐80 is a marsanidine analog with the highest I_1_R affinity among TCS analogs, with anpKi 6.04. In vivo pharmacodynamic assessment in anaesthetized Wistar rats demonstrated that intravenous administration of TCS‐80 at 100 µg/kg elicited a significant hypotensive response. Mean arterial pressure was reduced by −43.1 ± 5.1 mmHg, accompanied by a marked bradycardic effect of −112 ± 11 bpm, consistent with centrally mediated inhibition of sympathetic tone. The cardiovascular effects were significantly attenuated by both the mixed α_2_‐AR/I_1_R antagonist efaroxan and the α_2_‐AR selective antagonist RX82100, indicating coinvolvement of both receptor systems [36].

### 2.8 LNP Series

LNP series represents the first rationally designed family of I_1_R ligands devoid of significant α_2_‐AR. LNP 509, aminopyrroline analog of rilmenidine, demonstrated selective affinity for I_1_R (pK_i_ 6.2) while exhibiting negligible binding to α_2_‐ARs (pK_i_ < 5). LNP 509 (1 mg/kg, i.c.) induced a significant reduction in mean arterial pressure in anesthetized rabbits (from 93 ± 2–54 ± 3 mmHg), without potentiating reflex bradycardia, an α_2_‐AR side effect [37]. This finding underscores the therapeutic promise of I_1_R‐selective agents as safer antihypertensives. LNP 911 was radioiodinated, [^125^I]LNP 911, enabling high‐resolution binding studies and receptor mapping. In PC12 cell membranes, [^125^I]LNP 911 demonstrated nanomolar affinity and selectivity for I_1_R (K_D_ ≈ 1.4 nM; B_max_ ≈ 398 fmol/mg protein). Functionally, LNP 911 does not act as a direct agonist but instead serves as a positive allosteric modulator, enhancing the activity of endogenous and synthetic I_1_R agonists such as rilmenidine. This modulatory behavior is supported by findings showing that GIRK channel activation enhances [^125^I]LNP 911 binding, while inhibition reduces it, suggesting a dynamic interaction between I_1_R and GIRK channels [38]. LNP 906, a photoaffinity probe exhibited nanomolar affinity for I_1_R in PC12 cell membranes, while showing minimal affinity for I_2_Rs (pKi 3.88) and α_2_‐ARs (pK_i_ < 9). Binding was reversible and competitive in the dark, but UV irradiation (254 nm) induced irreversible, covalent receptor labeling, specifically at I_1_R, as evidenced by protection with rilmenidine but not rauwolscine. Functionally, LNP 906 acted as a selective I_1_R antagonist, blocking rilmenidine‐induced cAMP reduction without affecting melatonin responses and thus rendering it an essential tool for receptor mapping studies and the structural elucidation of I_1_Rs [39]. LNP 599 was developed as a third‐generation, centrally acting with selective I_1_R agonism (pK_i_ = 8.15 nM). In a high‐fat diet ‐induced rat model of metabolic syndrome, LNP 599 lowers systolic blood pressure and HR, reduces plasma catecholamines, and improves metabolic parameters, including cholesterol, triglycerides, nonesterified fatty acids levels and improves glucose tolerance. Importantly, LNP 599 also reversed microvascular rarefaction in skeletal muscle, improving both functional and structural capillary density. Crucially, the adiponectin‐stimulating effects of LNP 599 were abolished by the I_1_R antagonist efaroxan, confirming receptor specificity. These findings represent the first evidence of a selective I_1_R agonist restoring both cardiovascular and metabolic balance in metabolic syndrome [40, 41].

### 2.9 Agmatine

Agmatine is widely distributed in mammalian tissues, including the brain, heart, kidney, liver, adrenal glands, and intestine. At I_1_R, agmatine shows relatively high affinity (pK_i_ of 7.48 in human platelets) and a pKi of 6.15 in rat cerebral cortex membranes. At I_2_R, the affinity is markedly lower (K_i_ around 74 μM in human platelets). At α_2_‐AR, exhibits distinctly weaker binding, pK_i_ of 4.33at α_2A_, 3.79at α_2B_, and 4.58 at α_2C_ subtypes in recombinant human systems [42, 43, 44].

Agmatine acts as a centrally active ligand modulating neuroinflammation, neuroplasticity, and behavior through a broad I_1_R/I_2_R‐dependent mechanism [45, 46]. In AD mice injected with Aβ_1_–_42_, chronic agmatine administration normalized hippocampal TNF‐α, IL‐6, and BDNF levels [47, 48, 49]. Similarly, in the forced swim test, agmatine potentiated the antidepressant‐like responses of standard agents (e.g., SSRIs, bupropion), an effect reversed by I_1_R and I_2_R antagonists [50]. Agmatine restores neuroplasticity markers and normalize stress‐induced behavioral deficits in rodent models of insulin resistance and post‐traumatic stress disorder [51, 52].

Agmatine reduced depressive‐like states in ethanol withdrawal models (antagonized by efaroxan/idazoxan, potentiated by moxonidine/2‐BFI) and attenuated hyperlocomotion [53, 54, 55, 56, 57]. In opioid paradigms, agmatine blocked morphine place preference and sensitization, while enhancing analgesia and reducing tolerance (ED_50_ shift) confirming dual I_1_R/I_2_R mediated modulation of dependence and withdrawal [58, 59]. Agmatine shows significant antinociception via I_1_R/α_2_‐AR pathways, as confirmed in acetic acid writhing (blocked by efaroxan, not idazoxan) [60]. The guanidinium cation of agmatine, a privileged motif for imidazoline recognition is conserved in fentanyl‐guanidine hybrid derivatives [61] and included in the cyclic imidazoline system of 4(5)‐(2‐aminoethyl)imidazoline derivatives, constraining the flexibility of agmatine [62].

## 3 Pharmacology and Therapeutic Scope of I_2_R Ligands

The I_2_R, labeled by [^3^H]idazoxan, has emerged as a pharmacologically distinct entity primarily associated with mitochondrial monoamine oxidases (particularly MAO‐B) and glial‐expressed proteins such as creatine kinase [63, 64]. Functionally, I_2_R ligands exhibit a multi‐faceted pharmacological profile, characterized by neuroprotective, anti‐inflammatory, and opioid‐sparing effects. Their actions include the reversible inhibition of NMDA‐mediated Ca^2+^ influx [65], suppression of calpain activity [66], preservation of blood–brain barrier integrity, attenuation of glial activation, and downregulation of proinflammatory cytokines (TNF‐α, IL‐6) in both acute and chronic CNS injury models, potentiate opioid antinociception, delay tolerance, and in some cases modulate dependence‐related neurochemical adaptations [67]. Hypothermia in rodents serves as a robust in vivo assay for functional I_2_R agonism, while antidepressant‐like responses observed for compounds such as CR4056 and 2‐BFI are attributed to central monoamine modulation [68, 69]. Divergence in behavioral profiles among ligands like 2‐BFI, phenyzoline, and CR4056 in opioid synergy, drug discrimination, or neuroprotection supports the current hypothesis that I_2_R comprises a heterogeneous ensemble of binding proteins rather than a single receptor species.

**FIGURE 2:**
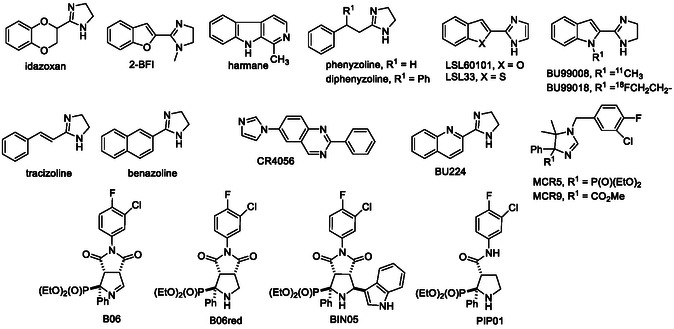
Representative I_2_R Ligands.

Selected I_2_R ligands are listed and the structure depicted in Figure 2.

### 3.1 Idazoxan

Idazoxan, was originally developed as a presynaptic α_2_‐AR antagonist and later became the first prototypical I_2_R ligand, endowed with antagonist I_2_R properties (pK_i_ of 7.98) over I_1_R (pK_i_ of 5.90), selectivity ratios of I_2_R/I_1_R = 119. Further refinement reveals subtype‐dependent binding at α_2_‐ARs, with idazoxan exhibiting antagonist potencies of pK_i_ = 7.73 ± 0.09 (α_2A_), 7.16 ± 0.10 (α_2B_), and 7.92 ± 0.08 (α_2C_) [70]. Its dual I_2_R/α_2_‐AR binding profile, despite modest selectivity, enabled early subclassification of imidazoline sites and guided the development of more selective I_2_R ligands. [^3^H]idazoxan has been further validated as radioligand in human postmortem and surgical brain tissue, as well as in glial tumor, demonstrating high‐affinity and saturable binding to a single class of sites with consistent K_d_ values across tissues, ranging from 10.8 nM in ependymomas to 23.6 nM in metastases. Importantly, no significant differences in affinity were observed between tumor and control tissues, indicating that idazoxan binds similarly to I_2_R across both normal and pathological samples [71]. Selectivity was ensured in these assays by inclusion of (–)‐adrenaline (5 µM) to block α_2_‐ARs, and non‐specific binding was determined using 100 µM naphazoline. In rat cortex, idazoxan binding was also unaffected by dexamethasone‐phenytoin treatment, confirming the stability of K_d_ (˜14–15 nM) and B_max_ values under neuroinflammatory conditions. Despite limited selectivity, [^3^H]‐idazoxan remains a key radioligand for I_2_R characterization, and continues to serve as a benchmark compound in IR pharmacology. Functionally, idazoxan acts as a reference antagonist in vivo, reversing I_2_R‐mediated potentiation of opioid analgesia (e.g., tracizoline, phenyzoline), and attenuating morphine tolerance while preserving neurofilament integrity. Despite this, it fails to potentiate acute analgesia in tail‐flick assays, suggesting an indirect glial mechanism of action. CNS studies show mixed neuroprotection: it is ineffective in NMDA‐induced astrotoxicity, yet reduces microglial reactivity and enhances astrocytic support in spinal EAE, consistent with a context‐dependent glial modulatory role. It lacks efficacy in antidepressant models, shows no 5‐HT_1a_ activity, and does not contribute to I_2_R‐mediated hyperphagia [72].

### 3.2 [2‐(2‐Benzofuranyl)‐2‐imidazoline] (2‐BFI)

[2‐(2‐Benzofuranyl)‐2‐imidazoline] (2‐BFI) [73] is a prototypical I_2_R ligand and radiolabeled [^3^H]‐2‐BFI is widely used to define I_2_R density and distribution in brain tissue due to its high affinity (pKi = 8.89), excellent selectivity over I_1_Rs (I_2_R/I_1_R = 4917) and α_2_‐ARs (I_2_R/α_2_AR = 2874), and minimal off‐target activity at μ‐opioid, adrenergic, or serotonergic receptors. Binding is preserved in MAO‐B‐deficient tissue, indicating that only a subset of I_2_R are MAO‐associated [74]. 2‐BFI exerts potent I_2_R‐mediated neuroprotection across ischemic, excitotoxic, and autoimmune models, attributed to glial modulation, MAO interaction, and mitochondrial I_2_R activation. In middle cerebral artery occlusion rats (3 mg/kg, i.p.), it significantly reduced infarct volume, preserved BBB architecture (occludin, ZO‐1 and collagen IV increase; MMP‐9 decrease), modulated apoptotic regulators (BCL‐2 increase and BAX decrease), and decreased TUNEL + cells. It inhibited NMDA‐induced Ca^2+^ influx via rapid, reversible, noncompetitive blockade of NMDA currents, and reversed EAE‐induced motor deficits while restoring B‐CK, CaATPase, and inhibiting calpain activity. In controlled cortical impact rats, repeated dosing (10 mg/kg, bid × 7d) elevated thermal and mechanical thresholds and downregulated spinal GFAP, Iba1, and TNF‐α; IL‐6, IL‐8, and TNF‐α suppression in glia‐neuron cocultures were neuron‐dependent. In diabetic ischemia, it elevated IL‐10, increased LC3‐II/p62 ratio, and stabilized lysosomal integrity [75, 76, 77, 78, 79]. Antinociceptive activity was confirmed in complete Freund's adjuvant (CFA), controlled cortical impact, and writhing models, with effects attenuated by pretreatment with idazoxan, considered I_2_R antagonist, confirming I_2_R mediation [80, 81]. Coadministration with opioids produced additive effects with fentanyl and oxycodone and synergistic enhancement with buprenorphine and NAQ [82]. Chronic 2‐BFI mitigated morphine tolerance and dependence, reducing naltrexone‐precipitated weight loss and suppressing locus coeruleus hyperactivity [83]. 2‐BFI is CNS penetrant and at 100–300 μmol/kg (s.c.) elicited antidepressant‐ and anxiolytic‐like effects in tail suspension test, forced swim test, and elevated plus maze, blocked by idazoxan, methysergide, and haloperidol [84]. Operant response suppression (ED_50_ ≈ 9.85 mg/kg) and potentiation of L‐DOPA‐induced circling in 6‐hydroxydopamine ‐lesioned rats indicate dopaminergic involvement. BU224, CR4056, phenyzoline, and harmane fully substituted for 2‐BFI in discrimination assays, consistent with shared I_2_R pharmacology [85]. At higher doses (10–40 mg/kg, i.p.), 2‐BFI induced sex‐dependent seizures unblocked by idazoxan, suggesting non‐I_2_R or idazoxan‐insensitive pathways [86]. It produced robust, dose‐dependent hypothermia (−3.56 ± 0.17°C at 32 mg/kg, i.p.), reversed by idazoxan but not efaroxan, WAY100135, or yohimbine. Locomotor activity remained unchanged at doses ≤10 mg/kg. D_2_ binding was weak (K_i_ ˜47 μM); D_1_ negligible [87]. Despite robust preclinical efficacy, 2‐BFI has not advanced clinically due to seizure liability, idazoxan‐insensitive toxicity, and inconsistent supra‐additive synergy (vs. CR4056, phenyzoline). Its analgesic efficacy is confined to chronic pain states, and pharmacokinetic properties remain suboptimal. Nevertheless, it remains the canonical I_2_R reference ligand and has informed the design of analogs such as BU224, BU99006, and tracizoline. Related heterocyclic 2‐(4,5‐dihydroimidazol‐2‐yl)benzimidazoles displayed a promising profile (pK_i_ I_2_R = 7.75; I_2_R /α_2_AR > 5649; pKa = 6.30) [88].

### 3.3 Phenyzoline

Phenyzoline binds the I_2_R with high affinity (pK_i_ = 8.60) and selectivity to both I_1_R (I_2_R/I_1_R = 1479). In contrast, diphenyzoline, the *ortho*‐phenyl‐substituted analog, displays reduced I_2_R affinity (pK_i_ = 6.80) and diminished selectivity (I_2_R/I_1_R = 40; I_2_ R/α_2_ = 45). Both ligands were inactive at all α_2_‐AR subtypes up to 10^−3^ M and displayed negligible μ‐opioid receptor binding (pK_i_ < 5 for phenyzoline; pK_i_ = 5.7 for diphenyzoline), excluding confounding orthosteric opioid activity and confirming I_2_R‐mediated pharmacology. Pharmacologically, phenyzoline acts as a potent I_2_R agonist. In murine antinociceptive assays (10 mg/kg, s.c.), it enhanced morphine‐induced analgesia by 60% (tail‐flick) and 46% (hot‐plate), effects reversed by idazoxan but not efaroxan or yohimbine, confirming I_2_R mediation. It also potentiated oxycodone antinociception in rats in a supra‐additive manner, outperforming 2‐BFI in synergy. Functional I_2_R agonism was corroborated by hypothermic response, and absence of α_2_‐AR interaction. Diphenyzoline, on the other hand, uniquely reduced morphine antinociception in tail‐flick (−41%) and hot‐plate (−20%) tests at 10 mg/kg s.c., an effect reversed by idazoxan but not efaroxan or yohimbine. It also amplified naloxone‐precipitated morphine withdrawal by 23%, suggesting negative modulation of I_2_R activity or inverse agonism. These data underscore diphenyzoline as the first I_2_R ligand to display functionally antagonistic properties in opioid assays, despite preserved I_2_R binding and structural similarity to phenyzoline [89]. Beyond their role in pain studies, these I_2_R‐selective ligands have been used to investigate IR interactions with dopaminergic signaling in the nucleus accumbens, suggesting broader neuropharmacological applications. Both ligands modulated dopamine efflux in a region‐ and receptor‐dependent manner, implicating I_2_R in mesolimbic neurotransmission and behavioral sensitization pathways. These findings extend the pharmacological relevance of I_2_R ligands beyond opioid tolerance and suggest their utility in studying reward‐related circuitry. Phenyzoline and diphenyzoline remain confined to preclinical research, with no evidence of progression to clinical trials [7, 90].

### 3.4 LSL60101 and LSL33

LSL60101 and LSL33, first reported by García‐Sevilla's group in 1995, LSL60101, exhibits high‐affinity binding to I_2_R in human brain membranes (pK_iH_ = 8.17 ± 0.19; K_i_ ≈ 0.676 nM) and exceptional selectivity over α_2_‐ARs (I_2_/α_2_ = 3,090), consistent with a two‐site binding model (high pKi (pKiH) and low pKi (pKiL) affinities for both binding sites) indicative of I_2_R heterogeneity (and favorable ADME profile. The design and synthesis of derivatives with different patterns of substitution led to a family submitted to 3D‐QSAR modeling incorporating in the study a broad ADMET [91]. Pharmacologically, LSL60101 induced dose‐dependent hypothermia in mice (–1.1 to –3.9°C at 50 mg/kg) and decreased hippocampal FADD protein expression by ˜34%, two established in vivo correlates of I_2_R engagement. In 5xFAD AD's mice, chronic administration significantly reduced Aβ plaque burden and p‐Tau levels (especially in combination with donepezil), and elevated synaptic markers PSD‐95 and synaptophysin. Antioxidant effects included decreased hippocampal expression of superoxide dismutase and glutathione peroxidase, along with downregulation of stress‐related ARE genes (Hmox1, Aldh2, iNOS) [92]. Transcriptomic profiling in the same model revealed downregulation of inflammatory genes (Cxcr2, Tlr5, Sele) and upregulation of chemokines (Cxcl10, Ccl12, Ccl8), suggesting I_2_R‐mediated modulation of glial and immune pathways [93]. Although LSL60101 showed no behavioral antidepressant‐like activity, this pharmacological dissociation reinforces the complexity of I_2_R signaling, where receptor binding affinity does not reliably predict mood‐related outcomes across all models [94, 95]. Pharmacomodulation of LSL60101 led to the bioisostere LSL33, which exhibits high‐affinity/selectivity to I_2_R in human brain membranes (pK_iH_ = 10.1 ± 0.57). Oral administration of LSL33 at 2 mg/kg for 4 weeks ameliorated 5XFAD cognitive impairment and synaptic plasticity, as well as reduced neuroinflammation markers [96].

### 3.5 Tracizoline and Benazoline

Tracizoline and benazoline, both conformationally constrained compared to phenyzoline, exhibit pK_i_ values of 8.74 and 9.07, respectively, and outstanding selectivity over adrenergic subtypes (tracizoline: I_2_R/α_2_AR = 7,762, I_2_R/α_1_AR = 2,344; benazoline: 18,621 and 2,691). 2‐BFI and tracizoline improve memory and reduce anxiety‐ and depression‐like behaviors in middle‐aged rats, suggesting potential therapeutic benefits for age‐related cognitive decline and mood disorders [97].

### 3.6 MCR5 and MCR9

MCR5 and MCR9 are the first reported I_2_R ligands lacking the nonsubstituted pattern in the imidazoline/imidazole nucleous, embodying a (2‐imidazolin‐4‐yl)phosphonate (MCR5) and a (2‐imidazolin‐4‐yl)carboxylate (MCR9). Incorporating a conserved N^1^‐(3‐chloro‐4‐fluorobenzyl) substitution and 5,5‐dimethyl groups, both ligands achieve high I_2_R binding affinity with favorable I_2_R/α_2_AR selectivity and excellent BBB permeability [98]. Their design represents a significant structural departure from classical arylimidazolines, expanding I_2_R chemical space while maintaining robust CNS exposure and in vivo neuroprotective efficacy in SAMP8 models of age‐related neurodegeneration. SAR efforts within the (2‐imidazolin‐4‐yl)phosphonate scaffold led to new members of the family with interesting affinities/selectivities. MCR5 is the most potent ligand in the family, with pK_i_ I_2_R = 9.42 ± 0.16, pK_i_ α_2_AR = 6.76 ± 0.22, and a balanced selectivity index of 457, far superior to that of idazoxan (pK_i_ I_2_R = 7.27). The methyl ester analog MCR9 has high I_2_R affinity (pK_i_ = 8.85 ± 0.21) and a fourfold improvement in selectivity (I_2_R/α_2_AR = 1862) [99].

MCR5 and MCR9 exhibited robust neuroprotective effects in SAMP8 mice, improving both short‐ and long‐term memory performance in the novel object recognition (NOR) test, increasing exploratory behavior in the open field test and the elevated plus maze, and reducing anxiety‐like traits, as indicated by decreased time spent in the closed arms of the elevated plus maze, collectively ameliorating BPSD‐like phenotypes [100]. Both ligands shifted APP processing toward nonamyloidogenic pathways: MCR5 decreased sAPPβ and increased ADAM10 and NEP expression, while MCR9 increased sAPPα with parallel upregulation of ADAM 10 and NEP. Antioxidant responses included decreased H_2_O_2_, decreased superoxide dismutase1, and increased Hmox1 expression in the hippocampus, alongside gene suppression of proinflammatory markers such as *Il‐1β* and *Tnf‐α*. MCR5 also reduced gene expression of *IL‐6* and *Cxcl10*. Synaptic plasticity was enhanced, as indicated by increased SYN protein levels for both ligands and increased PSD95 protein levels for MCR5. Apoptotic markers were consistently downregulated, including reductions in the p25/p35 ratio, Bax, caspase‐3, and spectrin breakdown products, indicating attenuation of both calpain‐ and caspase‐dependent pathways. Both compounds modulated survival‐related signaling pathways, including an increased ratio of phosphorylated AKT to total AKT (p‐AKT/AKT) and increased phosphorylation of GSK3β Ser9, which is associated with reduced phosphorylation of tau Ser396/404. In addition, both compounds decreased phosphorylation of ERK1/2 and reduced the ratio of phosphorylated cyclin‐dependent kinase 5 to total CDK5 (p‐CDK5/CDK5), collectively contributing to tau stabilization. Consistent with imidazoline I_2_R agonism, both compounds induced hypothermia. MCR5 reduced core body temperature by up to 2.2°C following acute dosing, with tolerance developing after repeated administration. MCR9 produced a milder hypothermic response, with a reduction of 2.3°C at a dose of 20 mg/kg administered intraperitoneally, and only partial tolerance over time. In the peripheral vasculature, MCR5 caused strong, concentration‐dependent vasorelaxation in isolated mouse aorta, surpassing the effects observed with the classical I_2_R ligand 2‐BFI. This vasodilation appeared largely independent of canonical I_2_R or α_2_‐AR mechanisms, as it was not blocked by antagonists such as idazoxan, BU224, or yohimbine. Instead, the effect was associated with inhibition of L‐type calcium channels, as indicated by reduced contraction in response to potassium chloride and Bay K8644. Partial involvement of potassium ATP dependent channels was suggested by attenuation with glibenclamide, while minor contributions were observed from K_v_ channels and the sodium/potassium ATPase, based on responses to 4‐aminopyridine and ouabain, respectively. Cycloxigenase inhibition partially reduced the vasodilatory effect, pointing to a role for prostacyclin, whereas nitric oxide synthase inhibition slightly enhanced the response. Importantly, MCR5‐induced vasorelaxation was maintained in aortic segments from aged mice with endothelial dysfunction, highlighting its potential therapeutic relevance in vascular aging [101].

### 3.7 B06, B06‐red, BIN05, and PIP01

B06, B06‐red, BIN05, and PIP01. B06 is a nonimidazoline, CNS‐permeant I_2_R ligand based on a bicyclic α‐iminophosphonate core with a 3‐chloro‐4‐fluorobenzyl substituent at the imine nitrogen [102, 103]. B06 binds with high affinity to I_2_R in human frontal cortex membranes with a pK_i_ = 8.56 ± 0.32 under a monophasic model. A biphasic fit revealed a high‐affinity component (pK_iH_ = 8.61 ± 0.28) accounting for ˜37% of total binding and a low‐affinity phase (pK_iL_ = 4.29 ± 0.20). At α_2_‐ARs, B06 exhibited moderate affinity (pK_i_ = 6.27 ± 0.56), yielding an I_2_R/α_2_AR selectivity ratio of ˜195, a profile superior to idazoxan (pK_i_ = 7.27 vs. 7.51) but lower than CR4056 (>117,000‐fold) or tracizoline (˜14,125‐fold). Functionally, B06 reversed cognitive and anxiety‐like deficits in both SAMP8 and 5xFAD Alzheimer's models at 5 mg/kg/day (oral, 4 weeks) [104]. It enhanced synaptic plasticity markers protein levels (increase in p‐CREB, PKAα and p‐AKT), suppressed tau hyperphosphorylation, and modulated apoptosis (decrease caspase‐3 protein levels and increase‐BAD protein levels) and inflammation (reduced GFAP protein levels and decreased Tnf‐α gene expression). In vitro, B06 protected SH‐SY5Y cells against 6‐hydroxydopamine ‐induced toxicity and reduced LPS‐triggered nitrite release in primary glial cultures, outperforming idazoxan and 2‐BFI. Reduction of the imine bond, yielding the saturated analog B06‐red, resulted in a high‐affinity ligand (pK_iH_ = 9.81 ± 0.21, 50% occupancy), yet selectivity dropped sharply (I_2_R/α_2_AR = 1.7), suggesting that the imine nitrogen contributes to α_2_‐AR discrimination. Despite this, B06‐red demonstrated cognitive improvement in *C. elegans* models and anti‐inflammatory activity in vitro. The addition of an indole moiety at the C‐imine position yielded BIN analogs with varying profiles. In particular, BIN05 maintained high affinity (pK_iH_ = 8.18, 21% occupancy) but lost α_2_AR selectivity and functionally displayed anti‐inflammatory activity in vitro and restored behavior in transgenic *C. elegans* AD strains [105]. The monocyclic phosphonate PIP01, generated via ring‐opening of B06, achieved outstanding affinity (pK_i_ = 9.98 ± 0.74; K_i_ = 1.05 nM) but lacked selectivity. Treatment with B06 improved cognition in SAMP8 mice and reduced capsaicin‐induced hypersensitivity without motor side effects. Target engagement was confirmed by reversal with idazoxan. ADME profiling demonstrated high Caco‐2 permeability, metabolic stability, and no hERG or CYP inhibition [106, 107]. Altogether, B06 and its analogs display potent neuroprotective and anti‐inflammatory activity across preclinical models. While modifications such as imine reduction (B06‐red) or ring opening (PIP01) enhance I_2_R affinity, they tend to compromise α_2_‐AR selectivity. Nevertheless, all compounds maintain favorable CNS‐relevant drug‐like properties and safety, supporting their continued development as I_2_R‐targeted therapeutics [108].

### 3.8 CR4056

CR4056 a 2‐phenyl‐6‐(1*H*‐imidazol‐1‐yl)quinazoline, is the first‐in‐class I_2_R ligand progressing in clinical trials with knee osteoarthritis [109, 110]. In rat brain membranes, CR4056 displaced [^3^H]2‐BFI with moderate potency (pK_i_ 6.22). Later studies in human cortex revealed biphasic binding: a high‐affinity component (pK_iH_ = 8.66 ± 0.43; 28%) and a lower‐affinity component (pK_iL_ = 5.12 ± 0.24), supporting the existence of multiple I_2_R conformers. CR4056 showed negligible binding to α_2_AR and opioid receptors and was inactive in WB4101‐, yohimbine‐, or naltrexone‐sensitive sites, confirming high pharmacological selectivity [111]. In drug discrimination studies in rats, the stimulus induced by CR4056 was fully mimicked by selected I_2_R ligands with non‐imidazoline structures, but not by BU224, clonidine, or morphine, thereby confirming a distinct I_2_R pharmacological profile. Oral administration of CR4056 attenuated inflammatory hyperalgesia in CFA‐induced pain (ED_50_ = 5.8 mg/kg; range: 6–60 mg/kg), reversed tactile allodynia in bortezomib‐induced neuropathy and streptozotocin‐induced diabetic neuropathy, and reduced postoperative mechanical hypersensitivity [112, 113]. Analgesic tolerance was absent after repeated dosing over 7–9 days and synergistic interactions with morphine were observed in both acute and chronic pain paradigms. Central effects include moderate hypothermia and robust discriminative stimulus properties, blocked by idazoxan and mimicked partially by 2‐BFI but not by BU224, morphine, or clonidine. CR4056 produced sex‐specific antidepressant‐like effects in forced swim tests, suggesting I_2_R‐mediated modulation of monoaminergic tone [95, 114]. Neuroprotective effects have been demonstrated in 5xFAD Alzheimer's disease models, where chronic treatment improved spatial memory, reduced blood–brain barrier disruption, and increased ApoE levels, indicating disease‐modifying potential. CR4056 acts as a reversible MAO‐A inhibitor, proposed to interact allosterically via mitochondrial I_2_R‐associated complexes [115]. Subchronic administration increased cortical and spinal noradrenaline concentrations, consistent with central noradrenergic activation. In primary sensory neurons, CR4056 inhibited PKCε phosphorylation and membrane translocation, a mechanism associated with its anti‐inflammatory analgesic properties [116].

### 3.9 [^11^C]BU99008

[^11^C]BU99008 a radiolabeled 2‐imidazoline–indole hybrid, constitutes the most extensively validated PET tracer for the I_2_R to date. In competitive binding assays using rat brain homogenates it displays high binding affinity (K_i_ = 1.4 nM) and preference for I_2_R over α_2_‐ARs (I_2_R/α_2_AR = 909), with no appreciable displacement observed upon incubation with α_2_AR antagonists or MAO inhibitors at saturating concentrations [117, 118]. In cortical tissue membranes [^11^C]BU99008, reveals a high‐affinity site (K_d_ = 1.3 ± 0.5 nM) and receptor density B_max_ = 255 ± 58 fmol/mg protein. In rodent studies, binding is prominently localized to the hypothalamus, thalamus, cortex, and brainstem, with 86% of total binding displaceable by BU224, confirming specific interaction with I_2_R. Notably, displacement in the brainstem was incomplete, suggesting a minor component of off‐target interaction, possibly related to mitochondrial MAO‐B–associated sites [119, 120]. In human PET studies, [^11^C]BU99008 displayed robust uptake with high volume‐of‐distribution values (e.g., V_T_,striatum = 105.7 ± 21.0 mL/cm^3^; V_T_,cerebellum = 41.9 ± 6.9 mL/cm^3^), consistent with known I_2_R regional densities. Pretreatment with idazoxan (80 mg p.o.) resulted in ˜60% signal reduction, while isocarboxazid had no significant effect, excluding MAO‐A/B contribution. Furthermore, PET imaging in early‐stage Parkinson's disease (PD) revealed a significant 52% increase in tracer uptake within the brainstem, with positive correlation to Unified Parkinson's Disease Rating Scale and Montreal Cognitive Assessment scores. These results establish [^11^C]BU99008 as a sensitive in vivo biomarker of reactive astrogliosis and a validated tool for quantifying I_2_R expression in neurodegenerative disease [118, 121, 122].

### 3.10 BU99018

BU99018 also referred to as FEBU, is the [^18^F]‐fluoroethyl analog of BU99008, developed to extend the imaging window and improve spatial resolution in PET applications by exploiting the favorable physical properties of fluorine‐18 (t_1_/_2_ = 110 min, lower positron energy). Affinity assays confirmed that BU99018 maintains high binding to the I_2_R, with a reported K_i_ of 2.6 nM, only modestly lower than that of BU99008. Selectivity over α_2_AR remains excellent, with an I_2_R /α_2_AR ratio of approximately 870, indicating minimal adrenergic interference. BU99018's binding is insensitive to MAO inhibitors and α_2_ AR antagonists, confirming target specificity. Pharmacologically, [^18^F]BU99018 exhibits favorable biodistribution and target engagement in preclinical PET studies. In rodents, it penetrates the blood–brain barrier effectively and accumulates in I_2_R enriched regions such as the cortex, hypothalamus, and thalamus. Pre‐administration of BU224 significantly reduced brain uptake, confirming target‐specific binding. No significant displacement was observed with MAO inhibitors, excluding off‐target interaction with mitochondrial MAO isoforms. Despite its promising preclinical profile, [^18^F]BU99018 has not yet undergone human PET evaluation. Its primary advantage lies in its compatibility with centralized radiopharmacies and wider clinical distribution due to the longer half‐life of ^18^F compared to ^11^C (110 min vs. 20 min). However, its chemical stability remains inferior to BU99008, and thus it has not replaced the latter as the lead clinical I_2_R tracer [123].

### 3.11 BU224

BU224 has become the benchmark antagonist for I_2_R studies. Binding studies in rat brain P_2_ membranes revealed a pK_i_ of 8.68 using [^3^H]2‐BFI, while subsequent assays in recombinant MAO‐B‐expressing membranes yielded pK_i_ = 8.01 [61, 124, 125]. Additional displacement assays using [^11^C]FTIMD also demonstrated consistent pK_i_ values (˜8.43), further validating its use in cross‐ligand PET competition protocols [126]. BU224 exhibits exceptional subtype selectivity: I_2_R/α_2_AR > 1000 and I_2_R/I_1_R = 832 and its interaction is not altered by α_2_‐AR antagonists or MAO inhibitors, reinforcing its binding independence from noradrenergic or mitochondrial monoamine targets.

Pharmacologically, BU224 functions as a low‐efficacy partial agonist or antagonist at I_2_R. It antagonizes 2‐BFI–induced effects such as antinociception and hypothermia (−2.18°C at 17.8 mg/kg), but does not potentiate opioid analgesia nor does it produce significant antidepressant‐like responses on its own [127, 128]. Autoradiographic studies using [^3^H]BU224 show dense binding in I_2_R rich regions such as the interpeduncular nucleus, area postrema, and ependymal zones, aligning with glial and astrocytic I_2_R expression patterns. In PET studies, BU224 reliably displaces radiolabeled tracers such as [^11^C]BU99008 and [^18^F]BU99018, demonstrating its essential role in tracer validation workflows.

## 4 Pharmacology and Therapeutic Scope of I_3_R Ligands

The I_3_R represents a functionally and pharmacologically discrete subclass of the IR family, with a primary physiological role in modulating glucose homeostasis via pancreatic β‐cell activation. I_3_R stimulation facilitates insulin secretion through inhibition of ATP‐sensitive potassium (K_ATP_) channels, leading to membrane depolarization, subsequent opening of voltage‐gated calcium channels (VGCCs), and sustained Ca^2+^ influx—culminating in insulin granule exocytosis. Unlike the better‐characterized I_1_R and I_2_R, I_3_R exhibit selective anatomical localization (predominantly β‐cells and brainstem nuclei) and distinct pharmacological signatures, including differential sensitivity to classical IR and α_2_‐AR ligands [129]. Mechanistic validation has been largely enabled by the selective antagonist KU14R, which consistently attenuates I_3_R‐driven responses in both in vitro and in vivo settings, thereby providing a reference standard for receptor assignment.

**FIGURE 3:**
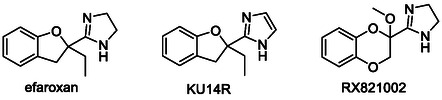
Representative I_3_R ligands.

Two selected I_3_R ligands are listed and the structure depicted in Figure 3.

### 4.1 Efaroxan

Efaroxan is a mixed α_2_‐AR and I_1_R antagonist (pK_i_ ≈ 7.5 at I_1_R), with demonstrated partial agonism at I_3_R. While not originally designed for I_3_R engagement, it produces insulinotropic effects via mechanisms analogous to glibenclamide, acting through K_ATP_ channel blockade and VGCC‐mediated Ca^2+^ influx. At 100 μM in MIN6 β‐cells, efaroxan increases cytosolic Ca^2+^ but fails to generate consistent oscillations. When present, oscillations are of low amplitude and reversible upon drug removal [130]. Insensitivity to dantrolene indicates a VGCC‐dependent pathway rather than RyR1‐mediated release. Notably, insulin release is enhanced but does not exceed glucose‐induced maximums, confirming a ceiling effect. In vivo, efaroxan (0.3 mg/kg, i.v.) reverses tizanidine‐induced spinal reflex depression, implicating central I_3_R involvement. Yohimbine, lacking imidazoline activity, fails to reverse the same effect.

### 4.2 KU14R

KU14R is the only pharmacologically validated selective I_3_R antagonist to date. While absolute binding affinities have not been published due to the lack of an I_3_R molecular clone, functional data across multiple models confirms high selectivity for I_3_R over I_1_R, I_2_R, and α_2_AR sites. KU14R (4–8 mg/kg, i.v.) blocks the hypoglycemic and insulinotropic effects of allantoin, canavanine, and morin in Wistar rats. In MIN6 and HIT‐T15 cells, KU14R dose‐dependently suppresses insulin release induced by I_3_‐active agents. Notably, KU14R (100 µM) attenuates harmane‐induced insulin secretion but does not prevent intracellular Ca^2+^ oscillations—indicating that I_3_R modulates exocytotic machinery rather than upstream Ca^2+^ dynamics. The compound's pharmacological resolution improves in combination with ryanodine receptor or PLC inhibitors (e.g., dantrolene, U73122), which allow dissection of receptor‐mediated vs. second‐messenger contributions to insulin release. KU14R thus functions as a gold‐standard tool for confirming I_3_R involvement in both endocrine and CNS models [131]. Efaroxan and KU14R share an analogous structure that differs only in the presence of an imidazoline ring or an imidazole ring, and the pharmacological outcome shifts from partial agonist activity to antagonist activity. This observation is indicative of the fact that the sensitivity of I_3_R to structural differences of the ligands is very high.

### 4.3 RX821002

RX821002 is a potent α_2_‐AR antagonist (pK_i_ ≈ 8–9 at α_2_) bearing an imidazoline ring, with negligible binding to I_1_R and I_2_R. Functional assays confirm selective antagonism at I_3_R sites, though direct radioligand binding data to I_3_R remains lacking due to the absence of an identified molecular target. The ligand's selectivity arises from its basic imidazoline group and hydrophobic aromatic ring system, yielding high CNS permeability and adequate engagement with supraspinal I_3_R. Pharmacologically, at 0.3 mg/kg (i.v.), RX821002 abolishes tizanidine‐induced MSR/DSR suppression in rats, without altering basal reflex tone. Yohimbine produces no comparable effect, underscoring RX821002's unique ability to antagonize I_3_R‐linked pathways. As such, RX821002 functions as a mechanistic probe distinguishing I_3_R‐mediated CNS suppression from α_2_‐AR inhibition [29].

## 5 Conclusion

The IR system, comprising I_1_R, I_2_R, and I_3_R subtypes, has evolved into a set of mechanistically distinct and therapeutically actionable targets. Since 2004, advances in ligand design, SAR elucidation, and receptor pharmacology have enabled selective modulation with clinical relevance.

I_1_R, involving Nischarin (IRAS) and potentially S1P_3_, regulates central sympathetic tone. Selective agonists such as moxonidine and rilmenidine (30–40‐fold α_2_‐AR selectivity) exhibit antihypertensive and neuroprotective effects. SAR studies across methylated imidazoline, thiazoline, and pyrroline scaffolds have defined structural determinants of I_1_R bias and α_2_‐AR avoidance. Functional studies highlight NF‐κB modulation, glutamatergic inhibition, and mitigation of Parkinsonian and Huntington‐like phenotypes.

I_2_R, a heterogeneous ensemble of MAO‐B–associated and glial proteins, is the most extensively characterized subtype. Ligands including 2‐BFI, CR4056, B06, and LSL60101 combine nanomolar affinity, CNS penetration, and α_2_‐AR inactivity. SAR analyses across benzofuran, quinazoline, iminophosphonate, and 2‐imidazoline cores reveal steric and electronic determinants of selectivity. Functional activation confers neuroprotection, anti‐inflammatory, and analgesic effects. PET ligands [^11^C]BU99008 and [^18^F]BU99018 (FEBU) validate I_2_R upregulation in neurodegeneration, supporting translational biomarker development.

I_3_R, localized to pancreatic β‐cells, modulates glucose‐dependent insulin secretion via KATP channel closure and Ca^2+^‐mediated exocytosis. Ligands such as efaroxan, RX821002, tizanidine, and the selective antagonist KU14R have delineated receptor function, confirming I_3_R specificity in β‐cell and CNS models.

Overall, IR pharmacology has progressed from receptor mapping to rational therapeutic exploitation. I_1_R ligands address cardiovascular and neurodegenerative disorders; I_2_R ligands provide neuroprotective and analgesic strategies; and I_3_R ligands offer mechanistically defined targets for diabetes (Table 1). Ongoing ligand optimization and receptor characterization promise the translation of these insights into clinical therapies.

**TABLE 1:** Summary of the main features of I_1_IR, I_2_IR, and I_3_IR mentioned.

Feature	I_1_IR	I_2_IR	I_3_IR
Proposed molecular targets	Nischarin/IRAS and sphingosine‐1‐phosphate receptor 3 (S1P_3_)	Mitochondrial MAO‐B, glial proteins (creatine kinase)	Pancreatic β‐cells and K_ATP_ channels
Key localization	Kidneys and platelets	CNS (glia), liver, and kidneys	Pancreatic β‐cells and brainstem nuclei
Therapeutic potential	Management of hypertension (sympathoinhibition), improves metabolic disorder parameters (glucose tolerance, lipid profile), and neuroprotection	Neuroprotection, chronic pain relief (analgesia, synergistic with opioids), and anti‐inflammatory effects	Diabetes management via regulation of glucose‐dependent insulin secretion.
Putative mechanism of action	Activation of the MAPK (p42/44) pathway and modulation of GIRK channels	Reversible inhibition of NMDA‐induced calcium influx and modulation of proinflammatory cytokines	Closure of K_ATP_ channels, leading to membrane depolarization and calcium influx
Partial/Mixed agonist ligands	Clonidine (benchmark ligand, dual I_1_IR/α_2_‐AR)	Efaroxan (Mixed α_2_‐AR/I_1_IR antagonist)	
Agonist ligands	Agmatine (endogenous I_1_IR with moderate affinity, antidepressant). Moxonidine and rilmenidine (central acting antihypertensive reducing side effects), and LNP 599 (improves metabolic syndrome parameters such as glucose tolerance and lipid profile) and lowers blood pressure)	2‐BFI (canonical agonist), CR4056 (analgesic, first‐in‐class agonist, clinical candidate), [^11^C]BU99008 (PET tracer for astrogliosis and neurodegeneration in Parkinson's disease) and [^18^F]BU99018 (long‐half life PET tracer), phenyzoline (hypothermic effects), LSL60101, LSL33, B06, MCR5, and MCR9 (improvement in the cognition of AD murine model, anti‐inflamatories, antioxidants)	Efaroxan (partial agonist, enhances insulin secretion)
Antagonist ligands	AGN192403, efaroxan, and LNP 906 (tool for receptor mapping)	Idazoxan, BU224 (benchmark antagonist/partial agonist, blocks 2‐BFI effects), diphenyzoline (first functional I_2_IR antagonist in opioid paradigms, reduces morphine analgesia)	KU14R (selective, pharmacological tool), RX821002 (also potent α_2_‐AR antagonist)

## Conflicts of Interest

The authors declare no conflicts of interest.

## Data Availability

Data sharing not applicable to this article as no datasets were generated or analyzed during the current study.
